# Role of Wnt Signaling in the Control of Adult Hippocampal Functioning in Health and Disease: Therapeutic Implications 

**DOI:** 10.2174/1570159X11311050001

**Published:** 2013-09

**Authors:** Abril Ortiz-Matamoros, Pamela Salcedo-Tello, Evangelina Avila-Muñoz, Angélica Zepeda, Clorinda Arias

**Affiliations:** Departamento de Medicina Genómica y Toxicología Ambiental, Instituto de Investigaciones Biomédicas, Universidad Nacional Autónoma de México, México D.F

**Keywords:** Alzheimer´s disease, Hippocampal plasticity, neurodegeneration, neurogenesis, neurorepair, Wnt signaling.

## Abstract

It is well recognized the role of the Wnt pathway in many developmental processes such as neuronal maturation, migration, neuronal connectivity and synaptic formation. Growing evidence is also demonstrating its function in the mature brain where is associated with modulation of axonal remodeling, dendrite outgrowth, synaptic activity, neurogenesis and behavioral plasticity. Proteins involved in Wnt signaling have been found expressed in the adult hippocampus suggesting that Wnt pathway plays a role in the hippocampal function through life. Indeed, Wnt ligands act locally to regulate neurogenesis, neuronal cell shape and pre- and postsynaptic assembly, events that are thought to underlie changes in synaptic function associated with long-term potentiation and with cognitive tasks such as learning and memory. Recent data have demonstrated the increased expression of the Wnt antagonist Dickkopf-1 (DKK1) in brains of Alzheimer´s disease (AD) patients suggesting that dysfunction of Wnt signaling could also contribute to AD pathology. We review here evidence of Wnt-associated molecules expression linked to physiological and pathological hippocampal functioning in the adult brain. The basic aspects of Wnt related mechanisms underlying hippocampal plasticity as well as evidence of how hippocampal dysfunction may rely on Wnt dysregulation is analyzed. This information would provide some clues about the possible therapeutic targets for developing treatments for neurodegenerative diseases associated with aberrant brain plasticity.

## INTRODUCTION

The mature brain undergoes continuous morphological changes in response to external and internal stimuli through the turnover and reorganization of neuronal networks and synapses [1-3]. These morphological adjustments determine the ability of neurons to incorporate new information from the internal and external environment and largely depend on the proper functioning of a variety of signaling pathways that control neuronal circuitry activity and neuronal shape. The secreted Wnt signaling proteins activate a variety of signaling pathways that modulate neuronal connectivity through downstream molecules involved in a number of physiological processes ranging from cellular morphology to gene expression [4]. All signaling events implicated in the Wnt pathway must occur in a coordinate fashion so synaptic contacts remain dynamic in the adult brain thus allowing a continuous fine balance between synaptic formation and synapse disassembly [5]. The hippocampus is a critical plastic brain region where significant network changes occur underlying a lifelong synaptic remodeling according to experience [3,6,7]. The hippocampus is particularly enriched in signaling molecules that influence many aspects of structural plasticity and network dynamics [8-11] besides being one of the two active neurogenic regions in the adult brain. The hippocampus is one of the most affected structures in pathological aging and emerging evidence has revealed a significant role of dysrupted Wnt signaling in the mechanisms of neuronal death and dysfunctional plasticity subserving neurodegenerative conditions such as AD [12] and frontotemporal dementia (FTD) [13].

### Wnt Signaling Pathways

Wnts are secreted cysteine-rich glycosylated and lipid modified proteins [14]. Palmitoylation at a conserved cysteine seems to be essential for Wnt signaling since removing the palmitate group or mutations in the cysteine residues result in loss of Wnt activity [15]. There are 19 Wnt genes identified in mammals, including human [16] with molecular sizes ranging from 39 kDa to 46 kDa [17]. Wnt signaling starts mainly by interaction of Wnt ligands to one of the 19 types of Frizzled (Fz) receptors. Wnt transduction pathways are complex and even when they have been studied as linear signaling cascades, it has been recently suggested that all Wnt pathways should be considered as part of an integrated cellular signaling network where different intra and extracellular stimuli converge [18]. The best characterized Wnt signaling is the canonical Wnt/β-catenin. In the absence of Wnt ligand, cytoplasmic β-catenin protein is degraded by the action of a complex composed of scaffolding axin protein, tumor suppressor adenomatous polyposis coli gene product (APC), casein kinase 1 (CK1), and glycogen synthase kinase 3 (GSK3) [19]. CK1 phosphorylates Ser45 of β-catenin priming the subsequent phosphorylation of GSK3 on Ser33/37/41. Phosphorylated β-catenins are recognized by the E3 ubiquitin ligase β-Trcp, which targets β-catenins for proteasomal degradation. Extracelular Wnt ligands bind to the seven-pass transmembrane receptor Fz and the low-density lipoprotein receptor-related protein 5/6 (LRP5/6) co-receptor. Wnt-Fz-LRP5/6 complex recruits the scaffolding protein dishevelled (Dvl) and axin on the plasma membrane leading to GSK3 inactivation. Without GSK3 phosphorylation, β-catenin accumulates in the cytoplasm and enters the nucleus, where it binds members of the lymphoid enhancer-binding factor/T cell-specific transcription factor (LEF/TCF) family and activates Wnt target genes expression [14]. Although the molecular mechanism of GSK3β inhibition is not completely understood, Wnt signaling has recently been reported to trigger the sequestration of GSK3β from the cytosol to multivesicular organelles, preventing its interaction with cytoplasmic substrates [20]. Another Wnt signaling pathway, the Wnt/PCP depends of Fz and Dvl to activate Rho GTPases and the Jun N-Terminal kinase (JNK). PCP components include Fz receptors, and the four pass trans-membrane protein Van Gogh like1 and 2 (Vangl1/2), Celsr1, 2 and 3, prickle and Dvl. Downstream these molecular elements, the PCP pathway acts through Rho and Rac small GTPases to control cytoskeleton remodeling and Jun N-Terminal kinase (JNK) to regulate gene expression [21]. Wnt ligands can also induce the release of calcium from intracellular stores, probably *via *heterotrimeric G-proteins in the so-call Wnt/Ca^2+^ pathway [22]. Wnt signaling through Fz receptor and Dvl mediates the activation of phospholipase C (PLC). PLC cleaves phosphatidylinositol 4,5-biphosphate (PIP2) into diacyl glycerol (DAG) and inositol 1,2,5-triphosphate (IP3). IP3 diffuses through the cytosol and interacts with endoplasmic reticulum calcium channels, resulting in the release of calcium ions and activation of the calcium calmodulin-dependent protein kinase II (CAMKII). DAG and calcium ions activate protein kinase C (PKC) [23]. PKC has several downstream intracellular targets including the nuclear transcription factors NFkB and cAMP responsive element-binding (CREB). CAMKII promotes the nuclear import of cytoplasmic protein nuclear factor associated with T cells (NFAT), which induces the expression of several genes.

**Fig. (1):**
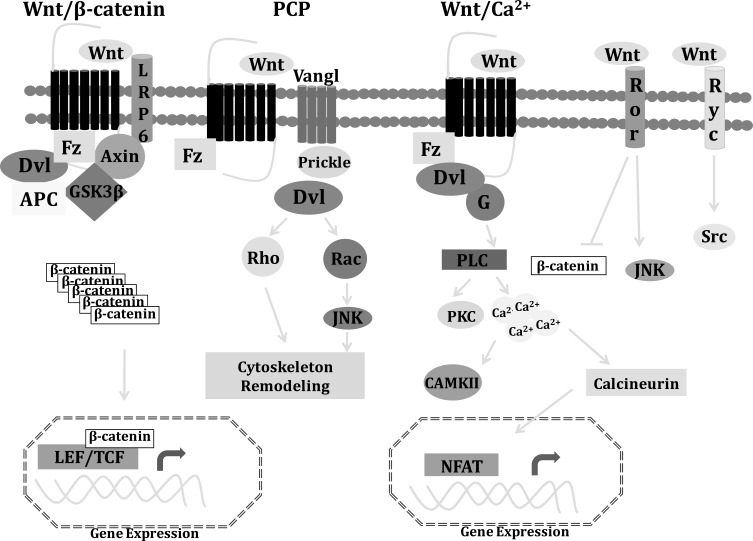
Schematic representation of Wnt signaling pathways. Wnt proteins activate several signaling pathways. APC, adenomatous polyposis coli; Dvl, Dishevelled; Fz, Frizzled; GSK-3β, glycogen synthase kinase-3β; LRP5/6, low density lipoprotein receptor related protein 5/6; CaMK, Ca2+/calmodulin dependent kinase; JNK, c-Jun N-terminal kinase; PKC, protein kinase C; Ror, receptor tyrosine kinase-like orphan receptor 1/2; Tcf/Lef, T-cell factor/lymphoid enhancer factor.

In addition to Fz receptors and LRP5/6, Wnt ligands can also bind to single pass transmembrane receptor tyrosine kinase (RTKs) of the Ryk and Ror families [24]. Wnt signaling through Ryk leads to the activation of Src proteins, and Wnt binding to Ror2 can inhibit β-catenin/TCF complexes and activate JNK [18]. Wnt4, Wnt5a and Wnt11 have been identified as activators of the non canonical Wnt pathways (Fig. **1**).

In the hippocampus several Wnt and Fz receptors have been found to be expressed throughout life. In the rat, Wnt4, Wnt5a, Wnt7a, Wnt8b and Wnt11 are present in hippocampal neurons from the embryonic stages to the adult stage [25,26]. Particularly Wnt7b expression remains in the DG blades and also outlines the pyramidal cell layer of CA3 in the adulthood [25]. Also, Fz3, Fz5 and Fz8 are expressed during hippocampal development [27] while Wnt3, Wnt5, and Wnt7a/b, Fz1, Fz2, Fz5, Fz8 and Fz9 increase during hippocampal synaptogenesis [27,28]. Fz9 has a selective expression pattern in the hippocampus and was found in both, neurons and astrocytes throughout life [29,30]. The specific pattern of genes encoding Wnt ligands, receptors, and inhibitory proteins reported in the adult hippocampus evidences the potential role for Wnt signaling in broad hippocampal functions. 

### Wnt Inhibitors

Wnt signaling is regulated by several types of secreted regulatory proteins, among the most well characterized are secreted Frizzled related protein (sFRP1 to sFRP5) [31,32], Dickkopf (DKK1 to DKK4) [33], and the Wnt-inhibitory factor 1 (WIF1) [34]. However, a variety of endogenous inhibitors such as Wise/SOST, Cerberus, IGFBP, Shisa, Waif1, APCDD1, and Tiki1 have also been described [reviewed in 35]. DKK1 interferes with Wnt canonical signaling preventing the binding of Wnt ligands to LRP5/6 co-receptors. WIF1 and sFRPs were initially found as Wnt scavengers that bind to Wnts and prevent Wnt-Fz activation [36]. Recently, sFRPs have been shown to act as counterparts of Wnts in gain-of- and loss-of-function experiments [reviewed in 37]. It should be noted that other functions of some sFRPs have been reported, such as the regulation of axon guidance by binding to Fz receptors [38]. It has been demonstrated that DKK1 induction is dependent upon induction by c-Jun [39] and p53, which is a sensor of DNA damage in cells [40]. 

### Wnt in the Hippocampus: From Development to Adult Plasticity

Various studies have shown a crucial participation of Wnt pathways at early stage of hippocampal development [41] and in fact, the expression pattern of Lef1 (a gene of the LEF1/TCF family of transcription factors) as well as other LEF1/TCF proteins are critical for the regulation of dentate gyrus granule cells generation and the entire hippocampal maturation [42,43]. Actually, the conditional inactivation of β-catenin in mice, results in an impairment of hippocampus development [44] and the inhibition of canonical Wnt signaling by DKK1 induces severe defects in the hippocampal structure [45]. Wnt proteins also exert influence on various features of neuronal circuit assembly through modifications of the neuroskeleton organization and synaptic assembly [4,5,46-53]. Studies in cultured hippocampal neurons have found that β-catenin are mediators of dendritic morphogenesis since the overexpression of a stabilized form of β-catenin leads to the development of a more complex dendritic arborization. In contrast, sequestration of β-catenin by overexpressing the intracellular domain of N-cadherin, decreases dendritic arborization and dendritic branch length. Moreover, the increased dendritic growth and arborization after high K^+^ depolarization depends on the intracellular contents of β-catenin and on the increase of Wnt activity since the number of dendritic branches decreases in DKK1-expressing neurons [54]. Interestingly, hippocampal dendritic arborization after depolarization depends on Wnt2 expression, which indeed belongs to the group of genes responsive to the transcription factor CREB, involved in plasticity events [55]. *In vitro* experiments in hippocampal neurons isolated from rats at embryonic day 18 have also shown a role for the non-canonical Wnt pathway function in dendritic arborization, in view that Wnt7b acting through Dvl1 increases dendritic branching by downstream activation of the Rac GTPase and the c-Jun N-terminal kinase (JNK) pathway. This effect is mimicked by Dvl1 overexpression and blocked by the Wnt antagonist sFRP, which is in line with the results from hippocampal neurons derived from a Dvl1 mutant mice [56]. Dvl1 is largely accumulated in developing axons where it directly regulates the function of the molecular complex PAR3-PAR6-aPKC (atypical protein kinase C) involved in axonal and dendritic differentiation in the hippocampus. The interaction of Dvl1 with aPKC resulted in its stabilization and activation of this atypical kinase. Additionally, treatment with conditioned media form cultured neurons expressing Wnt5a activates aPKC and promotes axonal differentiation. Together these results show that the effect of Wnt5a in the establishment of neuronal polarity depends on Dvl1-aPKC interaction [57] and demonstrates the critical role of Wnt during neuritic development. 

**Fig. (2):**
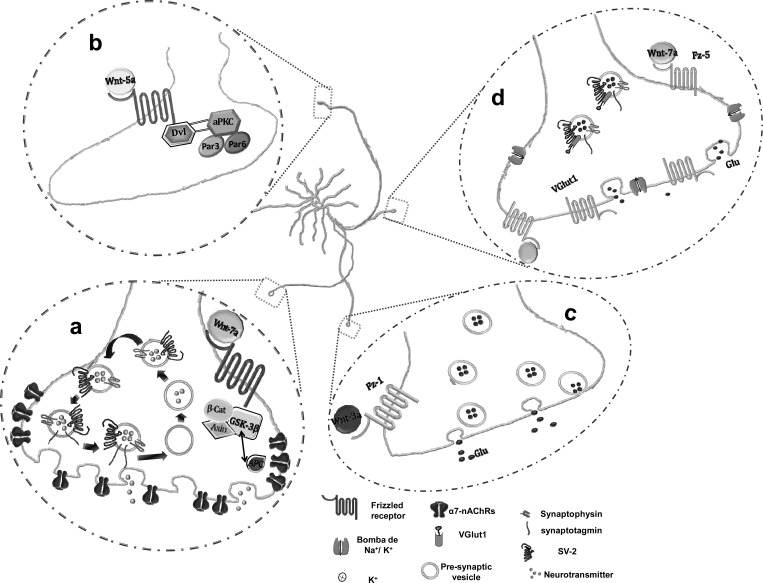
Role of Wnt signaling in the presynapsis. (a) Wnt-7a increases the clusters of presynaptic proteins (synaptophysin, synaptotagmin and SV-2), enhances spontaneous synaptic vesicles recycling and relocalizes α7-nAChRs. This last effect depends on APC dissociation from the β-catenin complex enabling the APC and α7-nAChRs interaction. (b) Wnt-5a promotes axon differentiation acting on Dvl which accumulates in developing axons and modulates the complex PAR3-PAR6-aPKC involved in neuronal polarization. (c) Wnt-3a binds to Fz-1 and increases excitatory presynaptic sites. (d) During depolarization, Wnt-7a modulates presynaptic components, inducing the clustering of Fz-5 with VGlut1.

Wnt signaling is also involved in presynaptic assembly and function. In cultured hippocampal neurons Wnt7a enhances the number of clusters of the presynaptic vesicle markers, synaptophysin, synaptotagmin and SV-2 through a mechanism independent of GSK3β activity and β-catenin stabilization in view that it does not require changes in Wnt-dependent gene expression. Moreover, administration of Wnt7a to hippocampal neurons induces spontaneous synaptic vesicle recycling and modulates the efficacy of synaptic vesicles exocytosis. These results point out the role of Wnt7a in the formation of new active sites for vesicle recycling and neurotransmitter release [26]. Other additional effect of Wnt7a on controlling neurotransmitter release seems to depend on its ability to relocalize nicotine acetylcholine receptors (α7-nAChRs) in presynaptic terminals. In hippocampal neurons Wnt7a induces the dissociation of APC from the β-catenin complex allowing the interaction between APC and α7-nAChRs [58,59]. As mentioned, Cerpa *et al*. [26] showed that Wnt7a decreases the paired pulse facilitation and increases the miniature excitatory post-synaptic currents (mEPSC) frequency enhancing neurotransmitter release at the CA3-CA1 synapses in hippocampal slices from adult rat. Similarly, Wnt3a rapidly increases mEPSC in embryonic hippocampal neurons depending on a fast influx of calcium ions from the extracellular space. Further, in this work the authors reported that the Wnt3a effects were also dependent on the presence of the LRP6 co-receptor suggesting a crosstalk between canonical and non-canonical Wnt signaling pathways [60]. In agreement with these findings the increased number of excitatory presynaptic sites elicited by Wnt3a was dependent on Fz1 activation in cultured neurons [61] (Fig. **2**).

There is evidence that emphasizes the involvement of Wnt receptors and ligands in the modulation of neuronal circuit assembly at pre- and post-synaptic levels. Depolarization of hippocampal neurons by high K^+^ induces the colocalization of Fz5 receptors with the pre- and post-synaptic markers, VGlut1 and PSD-95, respectively and mediates the effects of Wnt7a on synapse formation [28]. Recently, Wnt7a has been found to act also at a postsynaptic level promoting specifically the formation of excitatory synapses in hippocampal neurons. Even more, Wnt7a enhances dendritic spine density and maturity while Wnt7a-Dvl1 deficient mice show defects in spine morphogenesis and in mossy fiber-CA3 synaptic transmission [62]. 

**Fig. (3):**
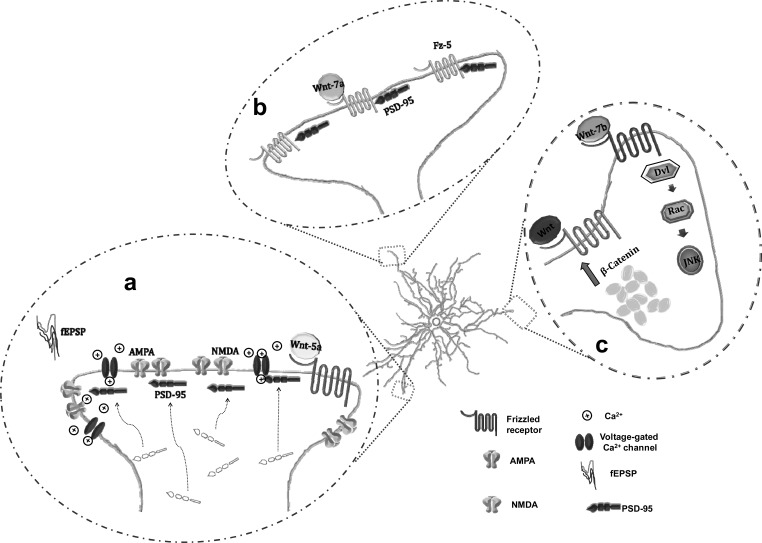
Role of Wnt signaling in the postsynapsis. (a) Wnt-5a augments fEPSP amplitude, promotes the recruitment of PSD-95 to dendritic spines, and modulates AMPA and NMDA responses. (b) Wnt-7a modulates postsynaptic components, inducing the clustering of Fz-5 with PSD-95. (c) The increase in β-catenin contents mediate dendritic morphogenesis. Wnt-7b augments dendritic branching acting through Dvl1 and downstream by Rac and JNK activation.

Interestingly, in hippocampal slices, Wnt5a enhances a calcium-dependent increase in the amplitude of field excitatory postsynaptic potentials (fEPSP). Besides, Wnt5a leads to short term changes in postsynaptic density protein-95 (PSD-95) distribution promoting its recruitment from a diffuse membrane pool to clusters in dendritic spines of mature hippocampal neurons. This effect has been attributed to a JNK1-dependent phosphorylation of PSD-95 on Ser295 [63]. Wnt5a modulates the activity of glutamatergic synaptic transmission increasing fEPSP amplitude at CA3-CA1 synapses dependent of both, AMPA and NMDA components of the excitatory postsynaptic currents (EPSCs) [64-67]. The ligand Wnt5a also affects inhibitory synapses in hippocampal neurons through its ability to induce GABA_A _receptors surface expression by promoting their insertion and clustering in the neuronal membrane. This effect enhances the efficacy of GABA synapses at the postsynaptic level as evidenced by a raise in the amplitude of GABA-currents and increasing GABA_A _receptors, effects mediated by CAMKII activation [68] (Fig. **3**). 

Long term exposure of cultured rat hippocampal neurons to Foxy-5 (formylated hexapeptide derived from the sequence of Wnt5a) that mimics the full action of the Wnt5a molecule, show the importance of this ligand in the mechanisms involved in neurite length promoting the membrane cluster of the SV2 (synaptic vesicle protein 2) and PSD-95 proteins. In addition, Foxy-5 increased mEPSC amplitude and frequency [69]. Therefore, Wnt5a appears to be a postsynaptic mediator of synaptic differentiation and plasticity in the hippocampus stimulating dendrite spine morphogenesis, inducing de novo dendritic spines formation, and increasing the size of the preexisting ones [70]. 

Besides the described Wnt effect on structural plasticity new evidence also suggests a role for the Wnt signaling in functional hippocampal events. It has been shown that in hippocampal brain slices inhibition of Wnt signaling impairs long-term potentiation (LTP) while activation of Wnt/β-catenin pathway facilitates LTP expression [71]. Additional evidence supports the idea that GSK3β inhibition is essential for LTP, since phosphorylation of the kinase at the inhibitory residue Ser9 is enhanced upon LTP induction in CA1 and dentate gyrus *in vivo*. Moreover, LTP is impaired in transgenic mice conditionally overexpressing GSK3β and this deficit is reversed by lithium treatment [72]. As it has been proposed that LTP might be the electrophysiological correlate of learning and memory, these results suggest that GSK3β is a key participant in these cognitive processes. The complexity of LTP expression may rely in the activity of several transduction pathways that act in concert to modulate diverse aspects of synaptic plasticity. In agreement, recent evidence shows that during the process of memory consolidation Notch signaling is transiently attenuated concomitantly with a transient increase in soluble β-catenin levels and GSK3β phosphorylation, indicating Wnt signaling activation in this event [73]. Another study reported that the Wnt-mediated suppression of GSK3β activity allows the activation of the mammalian target of rapamycin (mTOR) by strong synaptic activity, which is crucial for the induction of late phase of LTP and involves protein synthesis [74]. On the other hand previous studies revealed that GSK3β activation is required for memory reconsolidation in adult brain, as observed in Morris water maze performance of heterozygous GSK3β knockout mice impaired in their ability to form long-term memories [75].

Spatial learning has been associated with a selective increase of Wnt7 and Wnt5a levels in the hippocampus. The increase in Wnt7 levels was site and temporally specific since it was observed only in the granule cells of the dentate gyrus and expressed until 7 and 30 days after water maze [76]. Recently, studies on the relationships between Wnt/ β-catenin pathway and spatial memory have described that the expression of the calcium/calmodulin-dependent protein kinase type IV (CaMKIV) is modulated by Wnt3a and that the administration of lithium restores the levels of CaMKIV and improves the spatial memory deficits in a transgenic model of AD [77]. CaMKIV participates in the regulation of CREB-dependent genes involved in memory and neuronal survival [78, 79]. 

Recent evidence suggests that the Wnt pathway is not only an inductor of plastic events, but that it can be activated by synapse-dependent experience. Studies in animals exposed to an enriched environment (EE) have shown an enhancement of Wnt7a/b levels in postsynaptic CA3 pyramidal neurons. On the contrary, Wnt signaling inhibitor sFRP1 suppresses the increase on synapse numbers elicited by EE and reduces synapse numbers in control mice. Interestingly, Wnt7a/b application to CA3 neurons mimicks the effects of EE of synapse numbers, while eliciting excitatory activity in CA3 neurons elevates Wnt7a/b levels [3].

Altogether, the evidence highlights the importance of Wnt function in the regulation of hippocampal synaptic plasticity along life in events ranging from the appropriate neuronal circuit assembly, and the modulation of pre and postsynaptic terminals remodeling to the cognitive performance in experimental models.

### Wnt and Hippocampal Neurogenesis

Hippocampal neurogenesis takes place in the subgranular zone (SGZ) of the adult hippocampus, which constitutes a niche of stem and progenitor cell types that are continuously dividing and generating neurons that affect learning and memory [80]. The functional impact of new neurons on the existing neural circuitry and their contribution in hippocampal physiology under both healthy and pathological states has been demonstrated [reviewed in 81]. Each of the steps in neurogenesis is mediated by different signaling pathways, extracellular cues and cell-intrinsic mechanisms [10]. Wnt signaling and Wnt proteins play a critical role in stem cell self-renewal [82] as well as in the proliferation of the neural progenitor pool [83] and neuronal differentiation from neuronal precursor cells (NPCs) [84] in the developing central nervous system. Additionally, the canonical Wnt pathway has been shown to be involved in all different stages of adult neurogenesis in the SGZ [for review, see 85]. Evidence that Wnt signaling is a main pathway regulating neurogenesis comes from *in vivo* studies showing that in presence of the Wnt inhibitor sFRP2/3, there is a decrease in the percentage of adult hippocampal progenitors that differentiate into neurons. Furthermore, it has been shown that the orphan nuclear receptor Tlx activates Wnt/β-catenin signaling thus stimulating neural stem cell proliferation and self-renewal [86]. A recent work showed that Tlx can activate the expression of Wnt7a and the canonical Wnt/β-catenin pathway, suggesting that NSCs control their self-renewal in an autocrine manner [86]. In culture Wnt3 not only stimulates neuroblast proliferation but also instructs adult hippocampal progenitors to differentiate into neurons [87]. In particular, Wnt3a signaling has been shown to be essential for the normal growth of the hippocampus during development [41] whereas in adult neural stem cells, β-catenin that accumulates in response to Wnt3a induces the transcription of Neurod1 [88] a transcriptional factor that is essential for neuronal differentiation, maturation and survival [89]. Interestingly, Wnt3 protein levels and NeuroD1 mRNA levels decrease with aging along with a reduction in neurogenic differentiation of NPCs in the aged brain. However, *in vitro* the expression of receptors involved in Wnt signaling does not seem to be altered in the aged NSC [90].

Adult hippocampal astrocytes express Wnt family members like Wnt3 [87,90] and adult hippocampal progenitors express receptors for Wnts and other components of the Wnt/β-catenin signalling pathway [87], thus accumulating evidence suggests that a muticellular niche is needed for providing the required molecular signaling [87,91-93] necessary for neurogenesis to take place. Astrocytes have been shown to instruct differentiation of neural progenitor cells (NPCs) [90,94,95] and Wnts released by astrocytes have been shown to promote NPCs proliferation by inducing the expression of the mitotic regulator survivin [93]. 

Neurogenesis (in particular neuronal progenitor proliferation) has been shown to diminish during aging [96,97] along with the functional decline of hippocampal mediated learning and memory. In line with these observations, the experimentally induced decrease in neurogenesis has been positively correlated with impairment on long-term retention in different memory tasks [80]. Until lately, the target genes of Wnt/β-catenin signaling responsible for the different stages involved in adult neurogenesis had been scarcely identified. However, in a recent work Miranda *et al*. show that Wnt mediated signaling in the aged brain of mice led to a decrease of survivin expression in NPCs and to a diminished proliferation, while survivin protein levels increased after the activation of the canonical Wnt pathway in NPCs. Interestingly, the authors showed that the decrease in the neurogenesis rate in the aged brain relies on a deficit of NPCs in cell cycle progression dependent on the reduced levels of chromosomal pass aging protein survivin. Furthermore, it was suggested that a decrease in the TCF/LEF promoter activity occurs during aging and is dependent on the down regulation of Wnt genes [93].

So far, experimental evidence suggests that fine regulations in Wnt signaling, in particular in the canonical pathway differentially regulate aspects of neurogenesis thus promoting proliferation, differentiation an even survival of neurons [98, 99] and that during aging, Wnt signaling acting though genes such as NeuroD1 and survivin is modified thus altering different stages of the neurogenesis process. In line, it has been shown that the deletion of the tumor suppressor APC from a subset of NPCs leads to a modest decrease in cell proliferation in the DG of young adult mice, and to a reduction of neuroblasts. Thus suggesting that APC depleted NSC fails to pursue their normal differentiation pathway [100].

### Involvement of the Wnt Pathway in Neurodegenerative Diseases

Neurodegenerative diseases are generally associated to multiple neuronal abnormalities linked with changes at different levels of the structural and functional organization of neuronal networks, developmental defects or dysregulation of cellular signaling pathways that lead to synaptic atrophy and finally neuronal death. Altered Wnt signaling has been implicated in acute and chronic neuronal dysfunction associated to psychiatric conditions [101,102], ischemia [103,104], temporal lobe epilepsy [105], Alzheimer´s disease (AD) [12,106-110] and with some forms of frontotemporal dementia (FTD) [13].

The proposed relationship of altered Wnt signaling function with several psychiatric diseases is based on the evidence which shows that lithium has a positive effect on the treatment of the bipolar disorder symptom [111]. More recently various components of the Wnt pathway (e.g. Wnt2, Wnt7b and Fz9) have been found upregulated by chronic administration of antidepressant treatments. Particularly Wnt2 expression was reported to be highly elevated in the rat hippocampus, and the viral overexpression of Wnt2 was sufficient to produce antidepressant-like behavioral actions in an animal model of depression [112]. In this same line it has also been reported that chronic electroconvulsive sessions are accompanied by a CREB dependent increase of Fz6 mRNA levels in the granule cell layer of the dentate gyrus and in the CA3 region. Also, vector mediated inhibition of the Fz6 gene results in depressive like behavior in response to chronic unpredictable stress [113]. Thus, it is feasible to postulate that Wnt has a significant role in the modulation of highly complex emotional behaviors and its malfunctioning is implicated in the expression of some psychiatric symptoms.

Wnt antagonism seems also to take part in the induction of neuronal death as has been suggested by the fact that DKK1 levels are increased in the brain in different models of ischemia, excitotoxicity and exposure to amyloid-β peptide (Aβ) [reviewed in 114]. DKK1 is hardly detectable in the healthy brain, but it is strongly induced in brain tissue from AD patients or from patients with temporal lobe epilepsy and hippocampal sclerosis [109]. 

One of the neurodegenerative diseases in which Wnt signaling has been extensively documented is AD. More than a decade ago, it was suggested that sustained loss of function of Wnt signaling would determine the onset and development of AD [12]. Presenilin (PS) mutations that appeared in familiar AD were associated with altered intracellular trafficking and turnover of β-catenins as well as with down-regulated Wnt signaling [115,116].

Many other components of the Wnt signaling pathway have been implicated in the molecular pathology of AD. In Drosophila models of neurodegeneration, Wnt dysregulation can lead to neurofibrillary pathology [117] and the overexpression of Dvl1 increases the non-amyloidogenic α-secretase cleavage of APP [118] while DKK1-neutralizing antibodies are protective against synaptic loss in mouse models of AD [119]. Many of the pathological neuronal responses attributed to Wnt dysregulation in AD come from multiple evidences about the Wnt-dependent GSK3β activity regulation [reviewed in 120]. Experimental data suggest that GSK3β regulates APP processing [121,122] and prevents Aβ toxicity [123]. On the other hand, Aβ seems to interfere with the Wnt canonical pathway as well, leading to increased GSK3β function [109, 124], generating a vicious cycle that might exacerbate neuronal injury. Regarding to the canonical Wnt signaling pathway, the gene for LRP6 co-receptor has been identified as a risk factor for late-onset AD in ApoE4-negative individuals [125]. Interestingly, it has been reported that the Wnt pathway might be inhibited by ApoE protein, which likely binds to the coreceptor LRP5/6 [126]. Moreover, the ApoE4, implicated in sporadic AD [127] may activate GSK3β [128,129]. The canonical Wnt signaling inhibitor, DKK1 induces GSK3β-mediated tau phosphorylation in the hippocampus [130] as well as in cultured neurons [131]. Interestingly, DKK1 has been found elevated and colocalizing with neurofibrillary tangles and dystrophic neurites in degenerating neurons of AD brains [109]. Taken together, these evidences suggest that Wnt signaling might be a crucial pathological pathway that contributes to AD-related neurodegeneration and a link between amyloid and tau pathology.

Recent studies have found that the Wnt5a ligand and its receptor Fz5 were up-regulated in the brain of a mouse model of AD and in cultured cortical neurons by Aβ exposure [132]. Interestingly in this report the authors also found that Wnt5a signaling elicited the expression of proinflammatory cytokines such as interleukin 1β (IL-1β). 

Microglia plays an important role in inflammation and the hippocampus is densely populated with this type of cell. During aging and pathological conditions, the activated form of microglia has been found to be increased in the hippocampus [133,134] and the canonical Wnt signaling seems to have a role in microglia activation. Halleskog *et al*., [135] reported an increase in β-catenin expression in microglial cells that undergo proinflammatory morphogenic transformation in pathogenic neuroinflammation such as occurs in AD. In addition, in cultured microglia expressing both receptors, Fz and LRP5/6, Wnt3a can stabilize β-catenin and specifically enhance the expression of proinflammatory immune response genes that exacerbate the production and release of IL-6, IL-12 and tumor necrosis factor α (TNF-α). Given the significant role of neuro-inflammation in AD, the participation of Wnt in this process deserves to be analyzed in depth.

Recently, an elegant functional genomic analysis has shown a major role of Wnt dysregulation in brain samples from FTD associated with mutations in the progranulin gene and an important increase of the Fzd2 in a knockout progranulin gene mouse model [13]. 

Together, the above revised studies suggest that excessive and reduced Wnt function acting through the three signaling pathways may be involved at different steps of the neurodegenerative process in the adult brain. The specific disease outcome of such Wnt uncontrolled function may depend on the neuronal metabolic context and the cellular type (neurons, astrocytes or microglia) where Wnt signaling is exacerbated or inhibited. 

### Therapeutic Implications

An ideal compound with neuroprotective potential against hippocampal dysfunction should decrease or delay neuronal death, enhance neurogenesis, improve cognitive function, and control neuroinflammation. In view of the role played by Wnt pathways in maintaining neuronal homeostasis in the healthy hippocampus and their potential participation in brain disease it is feasible to suggest the targeting of Wnt signaling pathways to modify the progression of synaptic impairment, inflammation and ultimately neuronal death. In addition, the fact that Wnt signaling is involved in different steps of neural stem cell renewal suggests that purified Wnt proteins can be used as tools for cell replacement therapies in the treatment of neurodegenerative diseases and brain injury [86]. Adult neural stem cells are promising as therapeutic strategies given their potential to proliferate and differentiate into neurons and glia. On one hand, new neurons may incorporate into preexisting circuits, thus providing functionality to an altered circuit, but also, glial cells are part of the cellular niche needed for neuronal proliferation and survival. Among the different molecular signaling pathways for neurogenesis to occur, Wnt has shown to be a crucial one. It has been shown that following exercise in aged animals astrocytic Wnt3 levels increase thus re-stimulating different stages of neurogenesis [90]. Therapeutic strategies to increase Wnt positive effects may include the use of lithium which has been shown to enhance proliferation of adult hippocampal progenitors *in vitro *inducing them to become neurons at particular concentrations. Moreover, experiments in which lithium is administered to a mouse strain encoding a double-mutant form of APP, have shown to stimulate adult hippocampal progenitor cells proliferation and neuronal differentiation along with the improvement in cognitive functions through the inhibition of GSK3β and subsequent activation of Wnt/β-catenin signaling [136]. In a recent work, Matrisciano *et al*. [137] show that chronic mild restrain stress, which associates to hippocampal damage leads to an over expression of DKK1 levels and that inducing stress in a mice strain where DKK1 levels are reduced, not only diminishes neuronal loss, but increases neurogenesis and dendritic arborization [137]. 

Wnt signaling also mediates a positive neuron-astrocyte crosstalk for neuroprotection as has been found in mesencephalic neurons where Wnt1 effects depends on the astroglial response to oxidative stress and inflammation after neuronal injury, and requires Fz1 receptor and β-catenin stabilization to transmit pro-survival signals into the neuronal nucleus [138].

However to deep into the design of a molecule with Wnt positive effect, it should be considered that a fine balance between different Wnt ligands exists in the brain. For example, experimental models of behavior suggest that the inability of rats to tolerate stressful environments and the resulting anxiety may be associated with the enhanced expression of the Wnt2 gene and the resistance to the increase of Wnt7b levels in the ventral tegmental area [139] thus stressing the possibility that these ligands act in concert to promote brain adaptation when facing harmful situations. The functional reestablishment of a proper balance between different Wnt agonists and antagonist of the canonical Wnt pathway in anxiety as well as in major depression disorders may represent the biggest challenge to restore neuronal homeostasis in malfunctioning cells. 

Previous work has proposed that different canonical and non-canonical Wnt agonists, acting at different levels can be protective in AD [140]. Particularly the non-canonical Wnt activator, Wnt5a has a defending role against synaptic failure evoked by Aβ oligomers making this molecule a possible therapeutic target for AD therapy [65,67]. Likewise the search for compounds directed to neutralize the action of the Wnt inhibitors, e.g. DKK, may be a promising avenue for developing neuroprotective drugs for this devastating disease.

Given that Fz2 was found selectively elevated in the brain of patients with FTD and Fz2 activates a non-canonical Wnt pathway it is suggested that modulation of this pathway could be therapeutically beneficial for this demential illness.

However pharmacotherapy aimed at modulating Wnt pathways should consider the timing for application as well as the targeted cell type and brain region in order to provide specificity to the molecular processes underlying a specific neurodegenerative disease. For example, in terms of brain inflammation enhanced β-catenin signaling in microglia could be either beneficial or detrimental for the disease outcome. Also, Wnt activates proliferative pathways that may lead to uncontrolled cellular proliferation and tumor growth but as previously discussed may also represent a powerful therapeutic strategy for neuronal protection.

## CONCLUSIONS

Wnt signaling regulates many aspects of hippocampal development and continues critically involved in the adult regulating plasticity mechanisms. However the complexity of events that are under the control of the Wnt pathways poses the need to perform further studies that unravel the biological roles of the highly dynamic Wnt signaling through different stages of brain maturation as well as in disease. The expression of different genes and proteins of the Wnt signaling pathway in regions of the central nervous system that control learning and memory opens the possibility to understand how they can influence the processes of neuronal plasticity that are altered in dementias and neuropsychiatric disorders. Thus, new pharmacological developments can be specifically targeted at a particular disease entity to enhance treatment efficacy.
